# Evaluating associations of oxylipin metabolites and fatty acids with incident and prevalent type 2 diabetes in the Hispanic Community Health Study/Study of Latinos (HCHS/SOL)

**DOI:** 10.1371/journal.pone.0360656

**Published:** 2026-10-26

**Authors:** Carolina G. Downie, Heather M. Highland, Annie Green Howard, Mona Alotaibi, Tali Elfassy, Christina Cordero, Jennifer E. Below, Mercedes Carnethon, Susan Cheng, Martha Daviglus, Olga Garcia-Bedoya, Robert Kaplan, Tasneem Khambaty, Yilun Li, Adam G. Lilly, Dan-Yu Lin, Tao Long, Frank J. Penedo, Lauren E. Petty, Bharat Thyagarajan, Hsiao-Chuan Tien, Mohit Jain, Barrett M. Welch, Kari E. North, Christy L. Avery

**Affiliations:** 1 Department of Epidemiology, Gillings School of Global Public Health, University of North Carolina at Chapel Hill, Chapel Hill, North Carolina, United States of America; 2 Department of Epidemiology, Border Health Research Center, UTHealth Houston School of Public Health, Brownsville Campus, Brownsville, Texas, United States of America; 3 Department of Biostatistics, Gillings School of Global Public Health, University of North Carolina at Chapel Hill, Chapel Hill, North Carolina, United States of America; 4 Division of Pulmonary and Critical Care and Sleep Medicine, University of California San Diego, La Jolla, California, United States of America; 5 Department of Medicine, Division of Nephrology and Hypertension, University of Miami Miller School of Medicine, Miami, Florida, United States of America; 6 University of Miami Miller School of Medicine, Miami, Florida, United States of America; 7 Vanderbilt Genetics Institute, Vanderbilt University Medical Center, Nashville, Tennessee, United States of America; 8 Department of Medicine, Feinberg School of Medicine, Northwestern University, Chicago, Illinois, United States of America; 9 Department of Cardiology, Smidt Heart Institute, Cedars-Sinai Medical Center, Los Angeles, California, United States of America; 10 Institute for Minority Health Research, University of Illinois Chicago, Chicago, Illinois, United States of America; 11 Department of Medicine, University of Illinois College of Medicine at Chicago, Chicago, Illinois, United States of America; 12 Department of Epidemiology and Population Health, Albert Einstein College of Medicine, Bronx, New York, United States of America; 13 Department of Psychology, University of Maryland Baltimore County, Baltimore, Maryland, United States of America; 14 American Heart Association, Dallas, Texas, United States of America; 15 Carolina Population Center, University of North Carolina at Chapel Hill, Chapel Hill, North Carolina, United States of America; 16 Department of Sociology, University of North Carolina at Chapel Hill, Chapel Hill, North Carolina, United States of America; 17 Department of Biostatistics, Gillings School of Global Public Health, University of North Carolina at Chapel Hill, Chapel Hill, North Carolina, United States of America; 18 Sapient Bioanalytics, San Diego, California, United States of America; 19 Departments of Psychology and Medicine, University of Miami, Miami, Florida, United States of America; 20 Sylvester Comprehensive Cancer Center, University of Miami Miller School of Medicine, Miami, Florida, United States of America; 21 Vanderbilt Genetics Institute, Vanderbilt University Medical Center, Nashville, Tennessee, United States of America; 22 Department of Laboratory Medicine and Pathology, University of Minnesota, Minneapolis, Minnesota, United States of America; 23 Carolina Population Center, University of North Carolina at Chapel Hill, Chapel Hill, North Carolina, United States of America; 24 Sapient Bioanalytics, San Diego, California, United States of America; 25 School of Public Health, University of Nevada Reno, Reno, Nevada, United States of America; 26 Department of Epidemiology, Border Health Research Center, UTHealth Houston School of Public Health, Brownsville Campus, Brownsville, Texas, United States of America; Universita degli Strudi di Roma La Sapienza, ITALY

## Abstract

**Background:**

Inflammation is a common pathological feature underlying type 2 diabetes (T2D) risk and progression. Determining the roles of fatty acids and bioactive lipid oxylipin inflammatory mediators could inform T2D preventative efforts and therapeutic targets.

**Methods:**

Leveraging the Hispanic Community Health Study/Study of Latinos (HCHS/SOL), we evaluated cross-sectional and prospective associations of 81 oxylipins and polyunsaturated, monounsaturated, and saturated fatty acids with T2D. We assessed whether fatty acid levels differed at visit 2 by visit 1-prevalent T2D status and prioritized likely causal metabolites using Bayesian model averaging Mendelian randomization (MR-BMA).

**Results:**

Fifty-four fatty acids and oxylipins were associated with prevalent T2D at visit 1 (15,138 participants; 2,962 T2D cases), four of which (adrenic, dihomo-gamma-linolenic, tricosanoic, and lignoceric acids) were also associated with incident T2D by visit 2 (8,548 participants; 1,376 T2D events), though effects were attenuated for adrenic and dihomo-gamma-linolenic acids upon further adjustment for impaired glucose tolerance. Sixteen out of 28 fatty acids were significantly different at visit 2 between those with and without prevalent T2D at visit 1 (9,115 participants; 2,151 T2D cases), holding visit 1-measured fatty acid levels constant. MR-BMA prioritized one diHETrE oxylipin as a likely causal risk factor for T2D.

**Conclusions:**

Fatty acids and oxylipins showed complex patterns of association with T2D, including more associations likely reflecting overt disease processes in prevalent T2D, than incident risk-enhancing associations.

## Introduction

Inflammation is a key feature of metabolic dysregulation underlying the development of type 2 diabetes mellitus (T2D) [1–3]. The complex interplay of inflammation and metabolic processes, sometimes referred to as “metaflammation,” [4] involves multiple pathways and molecules, including free fatty acids [5]. A prevailing notion linking fatty acids, inflammation, and metabolic dysregulation is that individuals with obesity and T2D exhibit higher levels of free fatty acid levels due to overnutrition and increased lipolysis in excess adipose tissue [2,6]. This so-called “lipotoxicity,” [1] particularly from certain saturated fatty acids (SFAs), [5] may trigger inflammatory signaling and insulin resistance in multiple tissues, [2,5,6] although controversy over these mechanisms remains [7,8]. Other potential mechanistic links to beta cell dysfunction and T2D [9–11] include the role of primarily polyunsaturated fatty acid (PUFA)-derived [12,13] oxylipins as bioactive signaling molecules with both pro- and anti-inflammatory roles [13]. Thus, fatty acids across the saturation spectrum may contribute to T2D through both direct and indirect inflammatory effects [5]. A schematic summarizing several reported primary biosynthetic pathways of fatty acids and oxylipins [14–16] and their reported bioactive inflammatory roles in glycemic dysregulation [1,2,17,18] is shown in S1 Fig.

T2D and insulin resistance may also exacerbate dysregulated fatty acid metabolism and subsequent inflammatory signaling, resulting in fatty acid and oxylipin changes that co-occur with T2D development or in the context of disease progression [2,5]. For example, animal models have shown that insulin resistance and inflammation in adipose tissue promotes lipolysis and increases fatty acid levels [2,19]. Additionally, lipoxygenase (LOX)-derived oxylipins have also been linked with T2D complications, suggesting such pathways are altered by metabolic dysregulation in T2D [20]. Thus, the relationships between fatty acids, oxylipins, and T2D are complex and potentially bidirectional, complicating studies assessing the roles of fatty acids and oxylipin derivatives in T2D. Indeed, disentangling “cause rather than consequence” in metabolomics studies of cardiovascular and related diseases is a recognized challenge in epidemiology [21]. Most previous epidemiological studies have focused on the relationships of fatty acids and oxylipins with incident T2D, [22–26] but understanding the role of T2D on fatty acids and oxylipins could help improve understanding of disease manifestation and therapeutic targets. Leveraging the Hispanic Community Health Study/Study of Latinos (HCHS/SOL), a population with a high burden of diabetes, [27–29] we examined the relationships between oxylipins and fatty acids and T2D, including cross-sectional associations between oxylipins and fatty acids measured at study visit 1 and established T2D at study visit 1 (prevalent T2D), and prospective associations between oxylipins and fatty acid metabolites measured at study visit 1 and T2D diagnosed by study visit 2 among individuals free of T2D at baseline (incident T2D). Recognizing that pathologic changes may begin years-to-decades before overt T2D, and to account for unmeasured confounding and correlation between fatty acids and oxylipins, we applied Bayesian model averaging Mendelian randomization (MR-BMA) [30] to prioritize most likely causal fatty acids and oxylipins for T2D.

## Methods and materials

### Hispanic Community Health Study/Study of Latinos (HCHS/SOL)

HCHS/SOL is a multi-center, community-based cohort study of Hispanic/Latino adults in the United States designed to study the determinants and distributions of cardiovascular, lung, kidney, and liver diseases, among others [31]. A total of 16,415 participants of Mexican, Puerto Rican, Dominican, Cuban, and Central and South American backgrounds aged 18–74 years were recruited from four U.S. cities between 2008 and 2011: Chicago, IL; Miami, FL; Bronx, NY; and San Diego, CA [31]. Households were selected via two-stage sampling within census block groups [32]. At study baseline (visit 1), participants received standardized examinations and interviewer-administered questionnaires, and annual follow-up interviews were conducted via telephone to ascertain health information, including any diagnosis of diabetes. A second clinic examination visit was conducted between 2014 and 2017 (visit 2), and a third examination was conducted between 2020–2024 (visit 3). The HCHS/SOL was approved by institutional review boards at each study center as well as the HCHS/SOL Data Coordinating Center at the University of North Carolina at Chapel Hill and was conducted in accordance with the Declaration of Helsinki [31]. All participants gave written informed consent prior to the collection of any data [31]. Data for this analysis were accessed and analyzed between October 2022 (31/10/2026) and September 2026 (04/09/2026).

### Oxylipin profiling and annotation

Plasma oxylipin and fatty acid profiling from fasting blood samples collected at visits 1 and 2 was conducted in visit-specific batches by Sapient Bioanalytics using a non-targeted rapid resolution liquid chromatography-mass spectrometry (rLC-MS) methodology [33]. As has been described previously, [34,35] fatty acids and oxylipins were extracted from fasting plasma samples using rapid nano-valve switching and a three-pump system delivering a multi-tiered isocratic elution coupled to a high-resolution Agilent 6545B QToF mass spectrometer (Agilent Technologies, Santa Clara, CA, USA). Samples were then injected onto a custom packed, silica-based, mixed mode column allowing for reverse phase and weak ion pairing retention mechanisms. Samples were eluted using mobile phases starting with either water containing 0.1% acetic acid (positive mode) or 75:25 methanol:water containing 0.1% acetic acid (negative mode) and ending with 90:10 acetonitrile:water containing 0.1% acetic acid and 5mM ammonium acetate. Mass spectrometer parameters were set as: dry gas temp of 365 °C, dry gas flow rate of 13 L/min, nebuliser gas of 60 psi, sheath gas temp of 400 °C, sheath gas flow rate of 12 L/min, source voltage of (+)3500 / (-) 3000 V and nozzle voltage of (+) 50 / (-) 1000 V, mass range set to 50–1700 m/z, and data were collected at 8 spectra/s.

Data quality control (QC) was performed using a panel of isotopically labelled internal standards and interval pooled plasma samples to monitor fluctuations in instrument sensitivity, extraction efficiency, matrix artefact and mass accuracy [34,35]. To evaluate system suitability, mass calibration (passing a 5 ppm threshold) was performed prior to each plate run to assess mass accuracy, mass resolution, detector sensitivity and instrument cleanliness. For each plate run, QC was implemented through the following steps: isotopically labeled internal standards were added to each sample at the first preparation step to monitor matrix effects; bulk pre-aliquoted commercial pooled plasma (BioIVT) was placed in certain pre-defined wells and prepared identically to samples (internal bracket QC sample); bulk pre-aliquoted commercial pooled plasma was prepared external to the plate by hand (external bracket QC sample), and a preparation blank was prepared during each to assess background. The overall coefficient of variation was evaluated for the internal standards in the samples [34,35]. These internal standards and QC procedures support relative quantification and batch monitoring; absolute concentrations were not determined, and all oxylipin and fatty acid values reported in this study reflect relative abundance.

Oxylipins and fatty acids were annotated by aligning experimental accurate mass-to-charge ratio (m/z), retention time (RT) and MS2 spectra with an in-house curated library of standards as well as alignment with previously reported structural isomers [33]. These annotations often indicated multiple potential chemically related compound mappings, due to compounds with similar chemical formulas or m/z ratios co-eluting together. Oxylipin metabolites with chemical structures aligning with previously reported structural isomers for which biological pathways were unknown were annotated as “putative known oxylipins.” Oxylipins without database entries in LipidMaps or the Human Metabolome Database (https://hmdb.ca), but exhibiting spectral fragmentation patterns highly similar to those of known oxylipins were annotated as “putative novel oxylipins.” Their exact identities cannot be ascertained without further investigation, which is beyond the purview of this current work. Throughout this manuscript, we label oxylipins using a shorter name summarizing the class(es) of oxylipins to which these compound mappings belong. Full annotations and precursor fatty acid groupings of the 81 fatty acids and oxylipins are provided in S1 Table in S2 File. Several fatty acids and oxylipins were labeled with identical annotations and had similar M/Z and RT values but generally exhibited low to moderate correlations with each other (range: −0.11 to 0.70, see S2 Table in S2 File); therefore, we retained both metabolites for analyses.

### Alignment of oxylipins/fatty acids measured at visits 1 and 2

To identify pairs of matching metabolites measured at both study visits, we aligned the m/z and RT values. M/z values were compared by calculating $\frac{m/z\;visit\;\text{1}-m/z\;visit\;\text{2}}{m/z\;visit\;\text{1}}\;\times \;{\text{1}\text{0}}^{\text{6}}$ and RT values were compared by calculating RT visit 1−RT visit 2. Pairs with a m/z difference < 15 ppm and RT difference < 0.05 were considered aligned pairs; if there were multiple possible pairings, the pair with the smaller m/z difference, and then RT difference, was retained. Visit 1 and 2 values were imputed, winsorized, and centered and scaled as described above after defining the analysis sample of individuals with metabolites measured at both visits.

### Outcomes

T2D was defined by the following criteria: fasting plasma glucose ≥ 126 mg/dL (7 mmol/L), 2-hour postload glucose level (2-h oral glucose tolerance test [OGTT]) ≥ 200 mg/dL (11.2 mmol/L), or HbA1c level ≥ 6.5% (48 mmol/mol), self-reported diabetes diagnosis (excluding gestational diabetes), or self-reported use of antidiabetic medications [27]. Participants meeting these criteria at visit 1 (2008–2011) were classified as prevalent T2D, unless they were less than 30 years old or had a self-reported diabetes diagnosis age of less than 30, to exclude possible type 1 diabetes (T1D) cases, following previous studies identifying age 30 years as a discriminatory threshold for distinguishing T2D from T1D [36]. The same criteria were used to identify incident T2D by visit 2 among participants without prevalent T2D at visit 1. T2D-free individuals with fasting plasma glucose between 100–125 mg/dL (5.6–6.9 mmol/L) were categorized with impaired fasting glucose (IFG), and those with 2-hour post-OGTT levels between 140–199 mg/dL (7.8–11.0 mmol/L) were categorized with impaired glucose tolerance (IGT) [37]. Since OGTTs were not performed during the study visit 3 examination, incident T2D by visit 3 did not include OGTT ≥ 200 mg/dL as a criterion. Time-to-incident T2D was defined as the observed time between baseline and when T2D was first recorded (via annual follow-up, study visit 2, or study visit 3), or the time of last contact if the participant did not develop T2D.

### Covariates

For prevalent and incident T2D models, we adjusted for the following visit 1-measured covariates in our analyses: age, sex, Hispanic background group (Central American, Cuban, Dominican, Mexican, Puerto Rican, South American, more than one/other heritage), study center, waist circumference, physical activity (whether 2008 *Physical Activity Guidelines for Americans* moderate-to-vigorous aerobic activity threshold was met), anti-hypertensive, statin, and aspirin medication use, and the 2010 Alternative Healthy Eating Index (AHEI-2010) diet quality score [38]. We included age^2^, waist circumference^2^, and AHEI-2010 diet quality score^2^ terms in our models to account for possible non-linear effects of these continuous variables. Individuals missing relevant covariate data were excluded from analysis. In secondary analyses, we further adjusted incident T2D models for IFG or IGT. In sensitivity analyses, we also adjusted for current smoking status and triglycerides, and restricted analyses to non-users of statin, aspirin, and anti-hypertensive medications. Due to some missingness of these additional covariates in the primary analysis samples, to assess the influence of additional adjustment covariates, we calculated the difference in beta effect estimates between the additionally adjusted model and the original model within the same reduced analysis sample to limit differences due to sample size changes.

### Statistical analyses

Statistical analyses were performed using R (versions 4.4.0 and 4.5.0) [39]. We computed Spearman correlations of all visit 1-measured oxylipins and fatty acids in the prevalent T2D analytic sample, using the R “jtools” package (version 2.3.0) [40] to account for complex survey sampling weights. All descriptive statistics and association analyses were conducted using the R “survey” package (version 4.4−2) [41] to account for the complex survey design of the HCHS/SOL cohort. Median follow-up time for the incident T2D analytic sample was calculated using the reverse Kaplan Meier method [42,43]. We corrected for multiple testing using the Benjamini-Hochberg false discovery rate (FDR) procedure (q < 0.05) [44].

The longitudinal nature of HCHS/SOL enabled us to perform several analyses to assess the relationships between oxylipins and fatty acids and T2D. Fig 1 shows a schematic of analyses. First, we examined cross-sectional associations between visit 1-measured oxylipins and fatty acids and prevalent T2D (logistic regression). To maximize our sample sizes in the presence of participant attrition between study visits, as a primary analysis we examined associations between visit 1-measured oxylipins and fatty acids and incident T2D by visit 2 (Cox proportional hazards regression). In sensitivity analyses, we also assessed associations between visit 1-measured oxylipins and fatty acids and incident T2D by visit 3.

**Fig 1:**
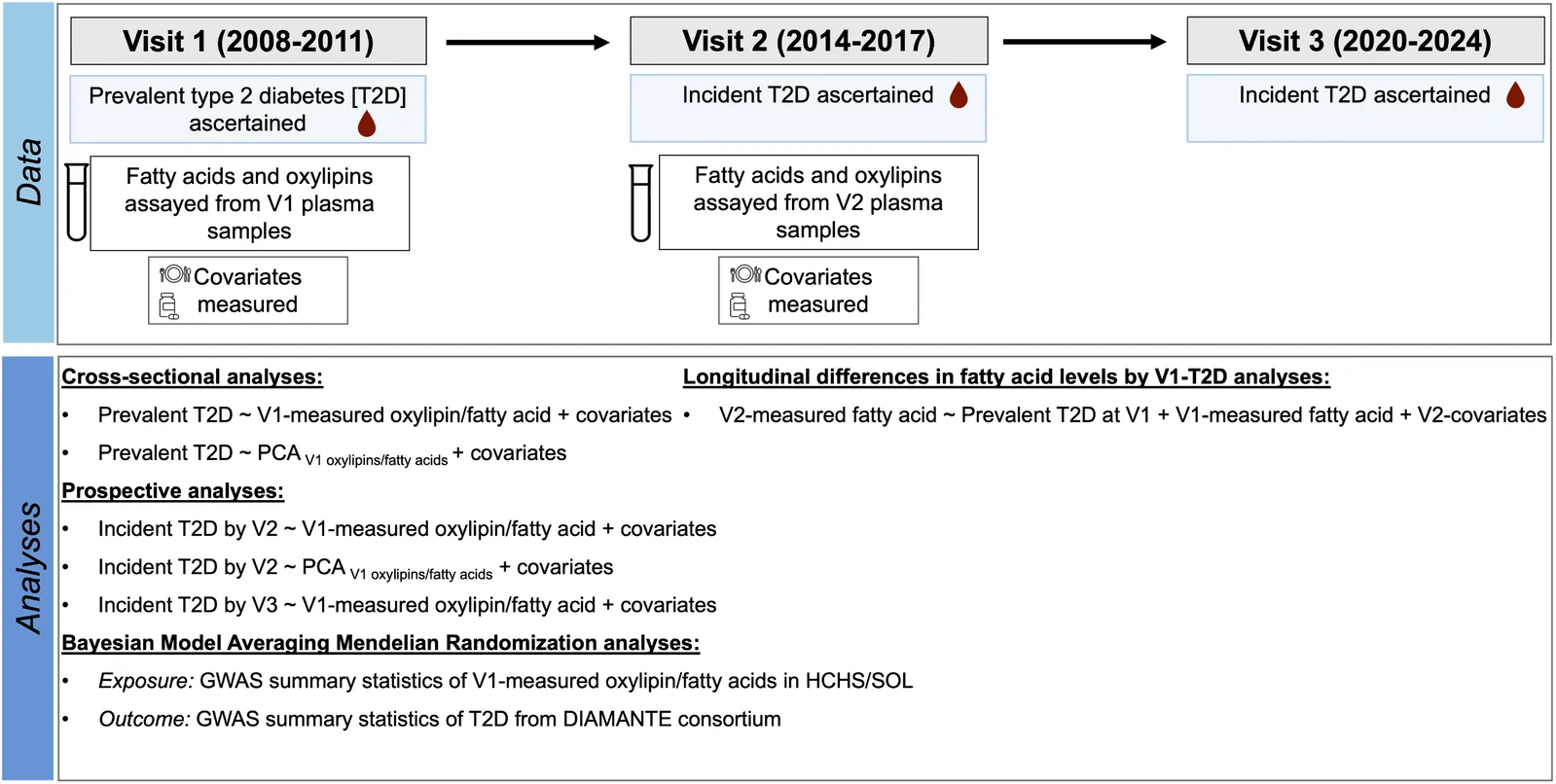
Schematic of data analysis approach.

We also conducted principal component analysis (PCA) with varimax rotation to assess whether patterns of fatty acids and oxylipins in aggregate were associated with T2D. PCs were calculated in the full sample of individuals with oxylipin and fatty acid data, separately for the prevalent and incident T2D analytic samples. Based on visual examination of the scree plot, percentage of variance explained by each PC, eigenvalues, and interpretability, we selected two PCs, explaining 30% and 11% of variation respectively, to reflect oxylipin and fatty acid patterns. We evaluated how these PCs associated with prevalent T2D and incident T2D by visit 2 in logistic regression models adjusting for the covariates described above, and years between visit 1 and visit 2 (for incident T2D). PC analyses were conducted in SAS (version 9.4, SAS Institute) [45].

Next, we sought to determine whether metabolite levels differed over time among those with prevalent T2D compared to those without T2D at visit 1. Because oxylipin annotations at both visit 1 and visit 2 indicated multiple potential chemically related compound mappings, we restricted to the 28 metabolites measured at both visit 1 and visit 2 annotated as single fatty acids. After excluding individuals without metabolites measured at both visits and those who developed incident T2D by visit 2, we used linear models to estimate associations between prevalent T2D at visit 1 and visit 2-measured fatty acid levels, controlling for fatty acid levels at visit 1, years between visit 1 and visit 2, and visit 2-measurements of the covariates described above, excluding covariates not measured at visit 2 (diet and physical activity).

Finally, recognizing the potentially biasing effects of unmeasured or residual confounding, correlations between metabolites, and the potential for oxylipins and fatty acid levels to change in the context of T2D development, we performed two sample Bayesian model averaging Mendelian randomization (MR-BMA) to prioritize likely causal fatty acids and oxylipins [30]. Our MR-BMA approach is described in detail in the **Supplementary Methods** in S1 File and S2 Fig.

## Results

### Descriptive statistics

After exclusions, there were n = 15,138 participants (T2D cases = 2,962 (19.6%), 47.4% male) in the prevalent T2D analytic sample, n = 8,548 participants (T2D events = 1,376 (16.1%), 48.5% male) in the incident T2D by visit 2 analytic sample, and n = 7,423 participants (T2D events = 2,137 (28.8%), 48.5% male) in the incident T2D by visit 3 analytic sample. Participants with prevalent T2D were on average older (mean age (SE) = 55.9 years (0.4)), had a higher waist circumference (mean waist circumference (SE) = 105.2 centimeters (0.4)), had higher triglycerides levels (mean (SE) = 178.6 mg/dL (5.0)), were less likely to be current cigarette smokers (survey-weighted percentage (SE) 17.0% (1.0)), and were much more likely to use anti-hypertensive medications (survey-weighted percentage (SE) 54.3% (1.4)), statins (32.4% (1.4)), and aspirin (40.6% (1.5)) than participants without prevalent T2D (**Table 1**). Similar trends were also observed for incident T2D by visit 2 (**Table 1**). Median follow-up time (interquartile range) to visit 2 was 5.96 years (5.51, 6.59 years) and 12.36 years (11.70, 13.13 years) to visit 3.

**Table 1:** Baseline characteristics of HCHS/SOL analysis samples.

	Prevalent T2D at Visit 1 analysis sample			Incident T2D by Visit 2 analysis sample		
	Total (N = 15138)	Prevalent T2D (N = 2962)	No T2D (N = 12176)	Total (N = 8548)	Incident T2D (N = 1376)	No T2D (N = 7172)
Age (years) (mean [SE])	41.2 (0.3)	55.9 (0.4)	38.8 (0.2)	45.1 (0.3)	53.1 (0.6)	44.0 (0.28)
Waist circumference (cm) (mean [SE])	97.3 (0.2)	105.2 (0.4)	96.0 (0.2)	96.1 (0.3)	102.9 (0.5)	95.1 (0.3)
AHEI diet score (mean [SE])	47.5 (0.2)	50.8 (0.3)	46.9 (0.2)	47.1 (0.2)	48.6 (0.3)	46.9 (0.2)
Gender = Male (%, [SE])	47.4 (0.6)	45.0 (1.4)	47.9 (0.66)	48.5 (0.8)	49.0 (1.9)	48.5 (0.8)
Current cigarette smokers (%, [SE])	20.8 (0.6)	17.0 (1.0)	21.5 (0.6)	20.4 (0.8)	19.3 (1.6)	20.1 (0.8)
Triglycerides (mg/dL)	132.9 (1.4)	178.6 (5.0)	125.3 (1.3)	125.2 (1.5)	160.1 (4.5)	120.3 (1.6)
Impaired fasting glucose (%, [SE])	--	--	--	20.7 (0.6)	47.7 (2.3)	16.9 (0.7)
Impaired glucose tolerance (%, [SE])	--	--	--	17.1 (0.6)	44.7 (2.3)	13.3 (0.5)
**Medication use** (%, [SE])						
Anti-hypertensives	17.1 (0.5)	54.3 (1.4)	10.9 (0.4)	11.0 (0.5)	25.1 (1.7)	8.9 (0.4)
Statins	8.1 (0.4)	32.4 (1.4)	4.1 (0.3)	4.0 (0.2)	8.5 (0.9)	3.4 (0.2)
Aspirin	18.5 (0.5)	40.6 (1.5)	14.9 (0.5)	14.8 (0.5)	19.6 (1.5)	14.1 (0.6)
**Physical activity** (%, [SE])						
High/medium physical activity level per 2008 physical activity guidelines	66.7 (0.6)	52.4 (1.4)	69.1 (0.7)	69.4 (0.8)	67.3 (1.9)	69.7 (0.9)
**Hispanic/Latino cultural background group** (%, [SE])						
Dominican	10.0 (0.7)	9.4 (1.0)	10.1 (0.7)	9.8 (0.7)	7.3 (1.0)	10.1 (0.8)
Central American	7.5 (0.6)	7.0 (0.6)	7.6 (0.6)	7.6 (0.7)	4.5 (0.6)	8.1 (0.8)
Cuban	20.7 (1.7)	23.4 (2.3)	20.3 (1.7)	20.3 (1.6)	20.0 (1.9)	20.3 (1.6)
Mexican	37.0 (1.7)	34.8 (2.1)	37.4 (1.7)	37.7 (1.7)	41.2 (2.3)	37.2 (1.7)
Puerto Rican	15.5 (0.8)	18.8 (1.2)	15.0 (0.8)	14.8 (0.8)	19.5 (1.9)	14.1 (0.8)
South American	5.2 (0.3)	3.8 (0.4)	5.4 (0.4)	5.2 (0.4)	3.0 (0.5)	5.5 (0.5)
More than one/other heritage	4.1 (0.3)	2.9 (0.7)	4.3 (0.3)	4.7 (0.4)	4.5 (1.0)	4.7 (0.4)

Sample size is unweighted; all other reported values are weighted to represent target populations. For percentages, SE values are also multiplied by 100.

Age for incident T2D analysis sample reflects mean age (SE) at study visit 2

For current cigarette smoking, n = 18 individuals in the prevalent T2D primary analysis sample and n = 10 individuals in the incident T2D primary analysis sample, respectively, had missing responses.

For triglycerides, n = 12 individuals in the prevalent T2D primary analysis sample and n = 8 individuals in the incident T2D primary analysis sample, respectively, had missing responses.

For impaired fasting glucose, n = 9 individuals in the incident T2D primary analysis sample had missing responses.

For impaired glucose tolerance, n = 374 individuals in the incident T2D primary analysis sample had missing responses.

### Metabolite missingness and correlations

The percent missingness for each of the 81 oxylipins and fatty acids measured at visit 1 ranged from 0% to 23.1%, though most metabolites (92.6%) were missing less than 2% of their values. This pattern was similar for metabolites measured at visit 2. The fatty acids and oxylipins showed varying correlations (ρ range: −0.27 to0.98, mean = 0.21) (S3 Fig). The strongest correlations were observed between fatty acids (ρ range = −0.22 to 0.90, mean = 0.34). For oxylipins, correlations varied both within and between precursor fatty acid groupings and were highest for oxylipins derived from docosahexaenoic acid (range: 0.03 to 0.76, mean = 0.27) and lowest for oxylipins derived from either arachidonic acid or eicosapentaenoic acid (range: −0.06 to 0.16, mean = 0.06).

### Metabolite associations with prevalent T2D

We identified 29 oxylipins and 25 fatty acids significantly associated with prevalent T2D (Fig 2, S4 Table in S2 File). Increasing relative abundance of nearly all oxylipins and fatty acids were associated with an increased odds of prevalent T2D, except for SFAs tricosanoic (m/z = 353.3428, RT = 0.459) and lignoceric (m/z = 367.3576, RT = 0.469) acids, which were associated with decreased odds of prevalent T2D (odds ratio (OR)_tricosanoic acid_ [95% CI per 1 SD increment] =0.88 [0.80, 0.96], FDR-corrected *p* = 7.37E-03; OR_ligonoceric acid_ [95% CI] = 0.77 [0.70, 0.86], FDR-corrected *p* = 2.83E-06). However, we note that another fatty acid with similar m/z and RT also annotated as lignoceric acid (m/z = 367.3577, RT = 0.496) had a significant positive association with prevalent T2D (OR [95% CI] = 1.09 [1.01, 1.17], FDR-corrected *p* = 3.20E-02). This positively-associated lignoceric acid metabolite was only modestly correlated (ρ = 0.26) with the other negatively-associated lignoceric acid metabolite (m/z = 367.3576, RT = 0.469).

**Fig 2:**
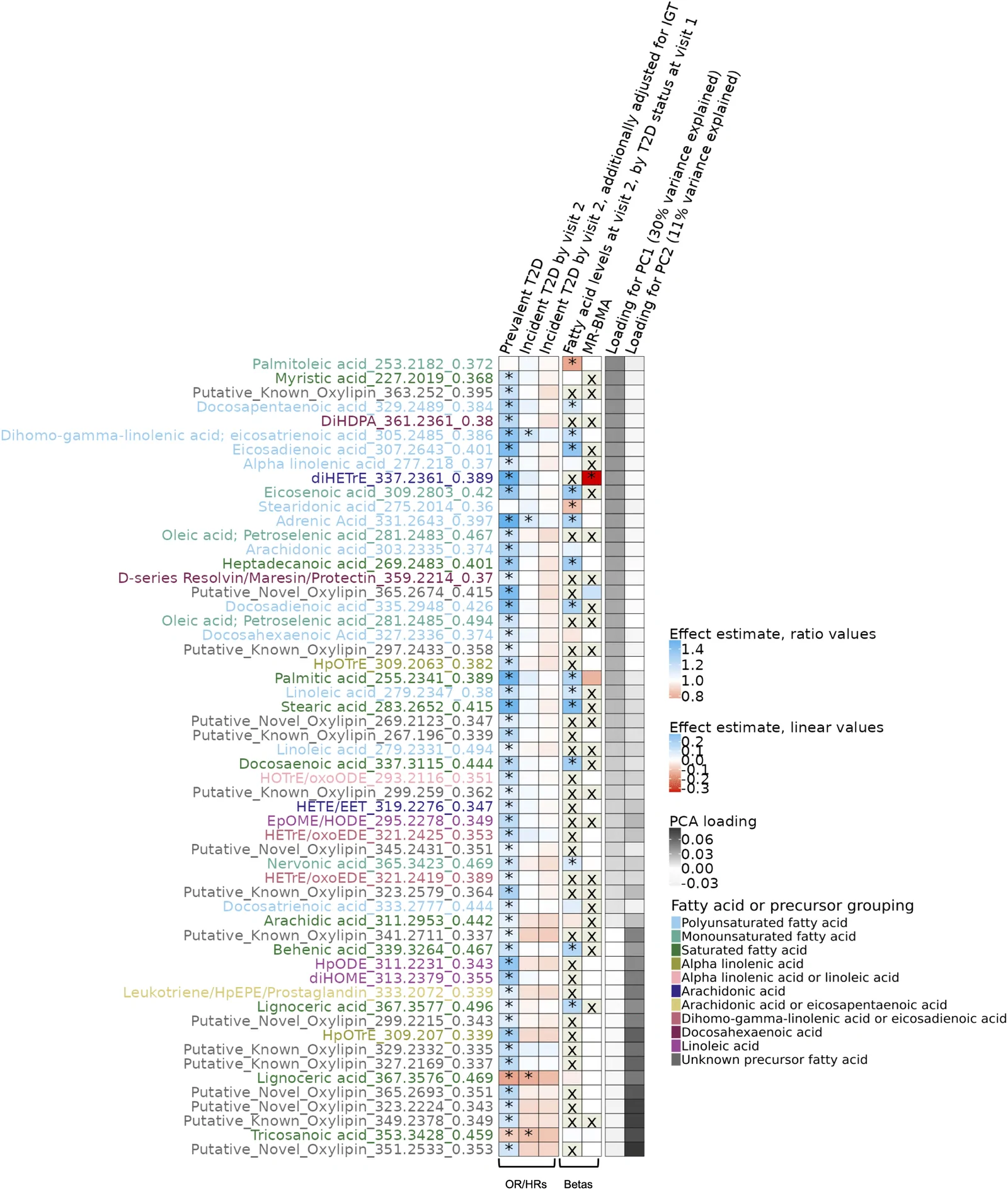
Heatmap of significant oxylipins and fatty acids across analyses. Fatty acids and oxylipins with at least one significant result across the individual-level analyses are shown; significance (p_FDR_ < 0.05) for a given analysis is indicated by “*”. Oxylipins and fatty acids not evaluated in an analysis are indicated by “X”.

In sensitivity analyses, effect estimates were consistent in models also adjusted for smoking status (median beta effect estimate difference across all 81 metabolites comparing the original model to a model also including current smoking status [interquartile range]= −2.93E-04 [−7.09E-04, 7.96E-05], sample size n = 15,120). Likewise, effect estimates were also generally consistent in models also adjusted for triglycerides (median beta effect estimate difference [IQR] comparing the original model to a model additionally adjusting for continuous triglycerides =  −3.41E-03 [−9.37E-03, 4.21E-03], sample size n = 15,126). When we restricted to individuals who did not report taking any of the three aspirin, statin, and anti-hypertensive medications (n = 9718, n prevalent T2D=958), effect estimates remained directionally consistent with small numerical changes (median effect estimate difference [IQR]=−2.27E-02 [−5.28E-02, 1.44E-02]), which is likely a result of the smaller sample size, since effect estimate differences were smaller in single medication-restricted analyses with sample sizes closer to the primary analysis sample (e.g., among non-statin users, the largest non-medication-restricted sample with n = 13,460, median effect estimate difference [IQR] = −5.34E-03 [−1.72E-02, 1.34E-02]).

Examining fatty acids and oxylipins in aggregate, both PC1 and PC2 were significantly and positively associated with prevalent T2D (OR per one-unit increment in PC1 [95% CI]= 1.37 [1.26, 1.48], *p* < 0.0001; OR per one-unit increment in PC2 [95% CI]=1.30 [1.18, 1.42], *p* < 0.0001). PUFAs and monounsaturated fatty acids (MUFAs) were most strongly loaded on PC1, along with several saturated fatty acids and oxylipins, whereas multiple SFAs and putative novel, known, and arachidonic-and linoleic acid-derived oxylipins were most strongly loaded on PC2 (Fig 2, S5a Table in S2 File).

### Differences in fatty acids levels at visit 2, by T2D status at visit 1

There were n = 9115 individuals (prevalent T2D cases at visit 1 = 2151 [23.6%], 48.0% male) with fatty acids measured at both study visits 1 and 2. After adjusting for visit 1 fatty acid levels, 16 of the 28 fatty acid levels at visit 2 differed between those with and without prevalent T2D at visit 1 (Fig 2, S6 Table in S2 File). Fourteen of these fatty acids were also associated with prevalent T2D at visit 1, with the same direction of effect (Fig 2). Neither stearidonic nor palmitoleic acids were significantly associated with prevalent T2D at study visit 1, but prevalent T2D was associated with decreases in the relative abundance of both metabolites at visit 2 (Beta_stearidonic acid_ [95% CI] = −0.105 [−0.184, −0.027]; Beta_palmitoleic acid_ [95% CI] = −0.152 [−0.227, −0.076]).

As in the prevalent T2D cross-sectional sensitivity analyses, additionally adjusting for current smoking status at visit 2 had minimal impacts on the effect estimates (median beta effect estimate difference [IQR] comparing the original model to a model also adjusting for smoking = 2.25E-03 [1.24E-04, 3.78E-03], n = 9,111, n = 1,331 current smokers). Similarly, additionally adjusting for triglycerides at visit 2 also had minimal impacts on results (median effect estimate difference [IQR] comparing the original model and a model additionally adjusted for continuous triglycerides = 1.59E-04 [−4.05E-03, 7.25E-03], n = 9,113, n prevalent T2D=2,150). When we restricted to individuals who did not report taking any aspirin, statin, and anti-hypertensive medications at visit 1 and also did not report taking any statin and anti-hypertensive medications at visit 2 (n = 4,601, n prevalent T2D=349), effect estimates remained directionally consistent, though with some modest numerical changes, again likely reflecting the smaller sample size (median effect estimate difference [IQR]=−3.73E-02 [−7.26E-02, −3.07E-03]).

### Metabolite associations with incident T2D

No oxylipins, but four fatty acids were significantly associated with incident T2D by study visit 2 (Fig 2, S7 Table in S2 File). PUFAs dihomo-gamma-linolenic acid (m/z = 305.2485, RT = 0.386) and its derivative adrenic acid (m/z = 331.2643, RT = 0.397) were significantly positively associated with incident T2D by visit 2 (hazards ratio (HR)_adrenic acid_ [95% CI per 1 SD increment] = 1.14 [1.06, 1.22], FDR-corrected *p* = 1.55E-02; HR_dihomo-gamma-linolenic acid_ [95% CI] = 1.16 [1.07, 1.26], FDR-corrected *p* = 9.00E-03). These fatty acids were also positively associated with prevalent T2D and exhibited positive differences at visit 2 for those with prevalent T2D at visit 1 compared to those without T2D (Fig 2). SFAs lignoceric (m/z = 367.3576, RT = 0.469) and tricosanoic acids (m/z = 353.3428, RT = 0.459) were significantly inversely associated with incident T2D (HR_lignoceric acid_ [95% CI] = 0.80 [0.71, 0.89], FDR-corrected *p* = 3.87E-03; HR_tricosanoic acid_ [95% CI] = 0.84 [0.75, 0.94], FDR-corrected *p* = 3.77E-02), consistent with the directions of effect for the associations between these saturated fatty acids and prevalent T2D. However, levels of these fatty acids at study visit 2 were not significantly different between those with and without prevalent T2D at visit 1. In sensitivity analyses, effect estimates were consistent in models also adjusted for smoking (median beta effect estimate difference [IQR] comparing the original model to a model also including current smoking status = 1.34E-03 [1.19E-04, 2.82E-03], sample size n = 8,539, n T2D events = 1,375). Effect estimates were also generally consistent in models adjusted for triglycerides (median beta effect estimate difference [IQR] comparing original model to model additionally adjusting for continuous triglycerides value = −2.48E-03 [−5.63E-03, 1.03E-03]; n = 8540, n T2D events = 1375). As in the prevalent T2D analyses, when we restricted to individuals who did not report taking any of the aspirin, statin, and anti-hypertensive medications (n = 5,958, n T2D events = 799), effect estimates remained generally directionally consistent with small numerical changes (median effect estimate difference [IQR]= −1.56E-02 [−3.87E-02, 9.54E-01]), again likely reflecting the lower sample size, as effect estimate differences were lower in single non-medication-restricted analyses with sample sizes closer to the primary analysis sample size (e.g., among non-statin users, n = 7,990, the largest single non-medication-restricted sample, median effect estimate difference [IQR]= −4.26E-04 [−7.28E-03, 9.55E-01]). No PCs were significantly associated with incident T2D by study visit 2 (PC1: OR [95% CI]=1.04 [0.93, 1.16], *p =* 0.46; PC2: OR [95% CI]=0.88 [0.76, 1.01], *p* = 0.07); PC loadings were similar to those in the prevalent T2D sample (S5b Table in S2 File).

Adjusting for IFG had minimal impacts on the effect estimates of these four fatty acids as well as the other metabolites (median beta effect estimate difference [IQR] across all 81 metabolites comparing the original model to a model additionally adjusting for IFG = 8.01E-03 [−2.62E-03, 1.07E-02]; n = 8539; n with IFG = 1920; n T2D events = 1375) (S7 Table in S2 File). In contrast, adjusting for IGT did attenuate the effect estimates for PUFAs adrenic acid (IGT-adjusted HR [95% CI]=1.05 [0.97, 1.13]) and dihomo-gamma-linolenic acid (IGT-adjusted HR [95% CI]=1.05 [0.96, 1.14]) (median effect estimate difference [IQR] across all 81 metabolites = 3.12E-02 [2.38E-03, 6.26E-02], n = 8,174; n with IGT = 1,838 with IGT; n T2D events = 1,289), (S7 Table in S2 File, Fig 2). However, effect estimates for SFAs lignoceric and tricosanoic acids remained more consistent with the original results (lignoceric acid: IGT-adjusted HR [95% CI]= 0.83 [0.74, 0.94]; tricosanoic acid: IGT-adjusted HR [95% CI]=0.85 [0.76, 0.96]).

In sensitivity analyses extending follow-up through study visit 3, effect estimates for these four fatty acids were attenuated compared to visit 2 analyses and no longer statistically significant (HR_adrenic acid_ [95% CI]=1.04 [0.98, 1.11], HR_dihomo-gamma-linolenic acid_ [95% CI]=1.06 [0.99, 1.13], HR_lignoceric acid_ [95% CI]=0.90 [0.82, 0.98], HR_tricosanoic acid_ [95% CI]=0.91 [0.83, 0.98]; median beta effect estimate difference comparing visit 2 primary analysis results to visit 3 results across all 81 metabolites [IQR]=1.78E-02 [−1.96E-02, 4.09E-02]; n = 7,423, n T2D events = 2,137 by visit 3) (S8 Table in S2 File). Additional adjustment for IGT in the visit 3 analyses resulted in additional modest attenuation, particularly for adrenic acid and dihomo-gamma-linolenic acid effect estimates (HR_adrenic acid_ [95% CI]= 0.99 [0.93, 1.06], HR_dihomo-gamma-linolenic acid_ [95% CI]=0.99 [0.92, 1.06]; median effect estimate difference comparing the original model for incident T2D by visit 3 to a model additionally adjusted for IGT, across all 81 metabolites [IQR] = 2.31E-02 [−7.85E-04, 4.97E-02]; n = 7,099, n = 1,754 with IGT, n T2D events = 2,036) (S8 Table in S2 File).

### MR-BMA

In MR-BMA analysis of n = 37 heritable oxylipins and fatty acids for which at least one genetic instrument could be identified, a diHETrE oxylipin (m/z = 337.2361, RT = 0.389) was prioritized as the top-ranked causal risk factor for T2D (margin inclusion probability = 0.601, *p* = 2.80E-04, FDR-corrected *p* = 1.04E-02, model averaged causal effect = −0.342) (Tables 2 and 3). This oxylipin was also significantly associated with prevalent T2D, though with the opposite direction of effect as reported for the model averaged effect of MR-BMA analyses (Fig 2) (OR [95% CI] per 1 SD difference = 1.51 [1.39, 1.64]), and was nominally significantly associated with incident T2D (HR [95% CI] per 1 SD increment = 1.10 [1.01, 1.21], *p* = 0.03, FDR-corrected *p* = 0.20). Sensitivity analyses with the *a priori* number of expected true causal risk factors ranging one to five also prioritized this diHETrE oxylipin as the top oxylipin or fatty acid, with similar rankings for the remaining top prioritized metabolites (S9 Table, S10 Table, S11 Table, S12 Table in S2 File). This diHETrE oxylipin exhibited modest to high correlation with all other oxylipins and fatty acids (mean = 0.31, range = −0.27 to 0.93). Of the other top ranked metabolites, palmitic acid (m/z = 255.2341, RT = 0.389) and a putative novel oxylipin (m/z = 365.2674, RT = 0.415) both exhibited modest average causal effects and were nominally (but not FDR-corrected) significant.

**Table 2:** Top 10 most likely models (MR-BMA for 37 risk factors, 26 instrumental variables).

Model	Posterior Probability	Causal effect
diHETrE_337.2361_0.389	0.211	−0.429
diHETrE_337.2361_0.389, Putative_Novel_Oxylipin_365.2674_0.415	0.102	−0.86, 0.529
Palmitic acid_255.2341_0.389	0.101	−0.396
Palmitic acid_255.2341_0.389, Putative_Novel_Oxylipin_365.2674_0.415	0.028	−0.816, 0.511
Heptadecanoic acid_269.2483_0.401	0.018	−0.329
diHETrE_337.2361_0.389, Heptadecanoic acid_269.2483_0.401	0.016	−0.693, 0.335
OPDA/OTrE_291.1969_0.347, diHETrE_337.2361_0.389	0.011	0.24, −0.414
Tricosanoic acid_353.3428_0.459	0.01	0.255
Palmitic acid_255.2341_0.389, diHETrE_337.2361_0.389	0.01	0.04, −0.467
diHETrE_337.2361_0.389, Tricosanoic acid_353.3428_0.459	0.008	−0.385, 0.162

**Table 3:** Top 10 most likely causal metabolites (MR-BMA for 37 risk factors, 26 instrumental variables).

Risk factor	Marginal inclusion probability	Model Averaged Causal Effect	Uncorrected *p* value	FDR-corrected *p* value
diHETrE_337.2361_0.389	0.601	−0.342	2.80E-04	1.04E-02
Palmitic acid_255.2341_0.389	0.28	−0.126	5.94E-03	1.07E-01
Putative_Novel_Oxylipin_365.2674_0.415	0.247	0.123	8.71E-03	1.07E-01
Heptadecanoic acid_269.2483_0.401	0.081	0	1.50E-01	9.97E-01
OPDA/OTrE_291.1969_0.347	0.055	0.014	4.69E-01	9.97E-01
Tricosanoic acid_353.3428_0.459	0.050	0.01	2.75E-01	9.97E-01
Eicosapentaenoic acid_301.2175_0.382	0.028	0.003	9.03E-01	9.97E-01
Heptadecaenoic acid_267.2334_0.382	0.027	−0.003	7.19E-01	9.97E-01
Docosahexaenoic Acid_327.2336_0.374	0.026	0.002	8.51E-01	9.97E-01
HpOTrE_309.2063_0.382	0.026	0.002	9.52E-01	9.97E-01

## Discussion

In this study, we leveraged cross-sectional, prospective, and longitudinal data to examine how oxylipins and fatty acids may affect T2D risk and in turn, may be affected by T2D. Although we identified few fatty acids and oxylipins associated with the risk of incident T2D, multiple individual oxylipins and fatty acids, metabolite profiles, and changes in fatty acid levels differed among those with and without prevalent T2D. This suggests that inflammatory dysregulation measured by fatty acids and oxylipins may be a hallmark of overt T2D, rather than only playing a risk-enhancing role.

To our knowledge, only one previous study has investigated the relationships between many oxylipins and incident T2D, conducted in the FINRISK cohort, a population-based sample of adults from Finland (~15 years of follow-up) [26]. In FINRISK, 76 out of 545 oxylipin metabolites were significantly associated with T2D, and a final oxylipin risk score was constructed of three independent oxylipins: 12-HHTrE, prostaglandin 8-iso-PGA1, and a novel oxylipin [26]. In contrast, in HCHS/SOL, we did not identify any oxylipins significantly associated with incident T2D by visit 2, or when extending follow-up to visit 3 (~12 years, more comparable to the FINRISK follow-up duration). However, in this FINRISK study, plasma samples were profiled using a different, eicosanoid-focused directed non-targeted LC-MS/chemical networking method (Sapient Bioanalytics) than the method used for our current study. Differences in chromatographic separation, ionization efficiency, and annotation/chemical-networking pipeline used to assign compound identities are thus plausible contributors to differences in the incident T2D-metabolite associations identified across these cohorts. Additionally, there are likely other demographic, social, and cultural differences, such as dietary patterns, between the FINRISK cohort and HCHS/SOL, contributing to our differing results despite efforts to account for these factors through covariate adjustment.

Our MR-BMA analysis significantly ranked a diHETrE oxylipin (m/z = 337.2361, RT = 0.389) (dihydroxyeicosatrienoic acid, also sometimes referred to as DHETs) as the top likely causal risk factor for T2D. Arachidonic acid-based epoxyeicosatrienoic acids (EETs) have strong, but short-lived anti-inflammatory effects due to their catalyzation via soluble epoxide hydrolase (sEH) into diHETrEs (sometimes called DHETs) [46]. Historically, diHETrEs themselves were believed to have weak or inactive effects, [47] though may reflect higher upstream EET production in response to inflammation. There is also some evidence that they have vasodilatory effects in animal models [48]. Thus, further investigation into their effects on T2D are necessary to confirm our findings.

This diHETrE was significantly positively associated with prevalent T2D and nominally positively associated with incident T2D but had a negative model average causal effect in MR-BMA. Similar observations of “flipped” directions of effect for metabolites in univariable versus multivariable MR models including other correlated metabolites have been reported for branched chain amino acids, [49] suggesting that properly accounting for potentially biasing effects of correlations between ‘omics risk factors can reveal more accurate effects of these risk factors. This may be in part what our results reflect, though a negative causal effect in the top ranked model in which this diHETrE metabolite was the only risk factor was also estimated. However, strong genetic instrumental variables were available for less than half of our characterized oxylipins and fatty acids, and these fatty acids and oxylipins are an incomplete subset of the known and yet-to-be characterized oxylipins in the human metabolome. Thus, it is possible that uncontrolled horizontal pleiotropy affected our MR-BMA analyses, which may also contribute to the flipped results.

Although we did not identify any oxylipins significantly associated with incident T2D, consistent with several previous studies, [23,24,50] we observed a significant positive association between PUFA dihomo-gamma-linolenic acid and incident T2D by study visit 2, as well as a positive association for adrenic acid. In line with previous findings, [51,52] we identified significant inverse associations between SFAs lignoceric and tricosanoic acids and incident T2D by visit 2. These fatty acids were also cross-sectionally associated with prevalent T2D with the same directions of effect, and adrenic and dihomo-gamma linolenic acids at visit 2 were also significantly increased for those with prevalent T2D at visit 1 compared to those without. Associations for lignoceric and tricosanoic acids were not substantially impacted by further adjustment for IFG or IGT. It has been hypothesized that very long-chain saturated fatty acids, including lignoceric and tricosanoic acids, may lower diabetes risk by lowering levels of palmitic acid-containing ceramides, lipid molecules that may contribute to insulin resistance [53].

In contrast, though minimally changed by IFG adjustment, the effect estimates of adrenic and dihomo-gamma linolenic acids were attenuated by adjustment for IGT. Evidence suggests that IFG and IGT reflect different pathological mechanisms of glucose dysregulation, [54] offering a plausible explanation for why these PUFAs were impacted by IGT, but not IFG, adjustment. Additionally, the fact that adrenic and dihomo-gamma acids were attenuated upon IGT adjustment, but tricosanoic and lignoceric acids were not majorly affected, suggests that these PUFA signals may reflect subclinical differences in glycemic regulation. Sensitivity analyses extending follow-up time for incident T2D to visit 3 also resulted in attenuated effect estimates before IGT adjustment (with modest additional attenuatation for the two PUFAs upon IGT adjustment). Collectively, these results support the hypothesis that these signals primarily reflect disease-related processes already underway prior to overt T2D diagnosis, rather than more risk-enhancing processes that occur years before overt disease onset.

Our cross-sectional and longitudinal analyses are also consistent with the idea that T2D has consequential impacts on oxylipin and fatty acid profiles. In contrast to the few associations identified in our incident T2D analyses, we identified 54 fatty acids and oxylipins cross-sectionally associated with prevalent T2D, most with positive effects, consistent with several previous small studies (n participants < 200) [5,55,56]. Positively associated oxylipins included several linoleic acid-derived epoxides (epOMEs) and diols (diHOME). EpOMEs and diHOMEs, particularly 12,13-diHOME, have been reported to be involved in biological functions with implications for cardiometabolic diseases, including inflammation and immune response, endocrine disruption, brown adipose tissue activation, and skeletal muscle regulation, though the potentially dose-dependent detrimental versus beneficial effects of these metabolites remains unclear [57].

The idea that insulin resistance, inflammation, and metabolic dysregulation are part of a “vicious cycle” [7] driven in part by increased free fatty acids levels in obesity is a prevailing model for T2D development [2,5,6]. However, a few researchers have pushed back on this paradigm, arguing that in the context of increased adiposity, fatty acid levels are in fact suppressed in a compensatory mechanism that results in ectopic fat deposition in other tissues, which ultimately drives the detrimental metabolic dysregulation apparent in obesity [7,8]. Because our metabolite data is measured via relative, rather than absolute, quantification, we cannot comment on the degree to which absolute fatty acid concentrations differ across individuals with and without T2D. Our cross-sectional and longitudinal analyses for prevalent T2D do suggest a shift in relative levels of fatty acids and oxylipins among those with T2D compared to those without T2D. Yet, we observed far fewer fatty acid and oxylipin associations with incident T2D, suggesting that the roles of fatty acids in the development of T2D through insulin resistance may be less prominent than previously hypothesized. However, delineating the mechanisms of impaired fat storage and its consequences on fatty acids, oxylipins, and metabolic dysregulation are beyond our scope.

This study has several limitations. Due to limitations in precise annotation of oxylipins, our assessment of whether metabolite levels at visit 2 differed among those with and without prevalent T2D at visit 1 was restricted to fatty acid measurements only. Though we adjusted for available medication use at visit 2 for these analyses, it is possible that this does not fully account for the potential impacts that changes in medication use between study visits 1 and 2 could have on fatty acid profiles. However, we also note that the prevalence of cardiovascular-related medication use in Hispanic/Latinos and in the HCHS/SOL specifically is low compared to national estimates [58,59]. Biological pathways of many of the significant oxylipins we identified (labeled as “putative novel/known oxylipins”) remain unknown, limiting biologically-informed interpretations of our findings. Furthermore, we could not formally replicate our findings in an independent dataset, as a set of the same or harmonized metabolites are not currently available in other cohorts. This is an ongoing challenge, particularly for untargeted metabolomics studies in which full annotations are not available for all metabolites. Moreover, although MR-BMA enabled prioritizing causal risk factors, we could not include all fatty acids and oxylipins due to lack of strong genetic instruments. Finally, participant attrition at subsequent study visits over the course of longitudinal follow-up resulted in progressively smaller sample sizes at study visits 2 and 3, limiting power to detect any associations with small effects.

## Conclusions

In one of the largest studies of fatty acids and oxylipins and T2D to-date, we used cross-sectional and prospective individual models, PCA, and MR-BMA to evaluate the complex relationships between fatty acids and oxylipins and T2D. Our results suggest that fatty acid and oxylipin profiles primarily reflect subclinical and overt disease processes in the presence of T2D, with likely more limited risk-enhancing roles for these metabolites. Future studies should conduct similar analyses in other diverse populations to improve understanding of the generalizability of our results across populations. Furthermore, future efforts to annotate and characterize these oxylipin metabolites, particularly putatively novel oxylipins, will be important to better understand the biological pathways and disease processes to which these metabolites may contribute.

## Supporting information

S1 File Supplementary Methods. (DOCX)

S2 File Supplementary Tables. (XLSX)

S1 Fig Primary biosynthetic pathways of n-6 and n-3 polyunsaturated, and saturated and monounsaturated fatty acids, and oxylipin metabolites, and conceptualized bioactive inflammatory effects on glycemic dysregulation. In fatty acid biosynthesis pathways, fatty acids are bolded and solid arrows between them indicate relationships between each fatty acid; dashed arrows indicate enzymatic reactions yielding oxylipin metabolites from these precursor fatty acids. Dotted arrows illustrate the stress-inducing effects that excess levels of free fatty acids have been reported to have on adipose tissue and pancreatic beta cells; subsequent inflammation and insulin resistance can further lead to release of fatty acids. Gray arrows indicate the reported effects on inflammation: adipose tissue release inflammatory molecules (e.g., cytokines, chemokines), triggering recruitment of macrophages and other immune cells, contributing to additional inflammation, thus promoting inflammation in islet cells and other tissues and leading to the production and release of additional inflammatory molecules. Oxylipins are believed to contribute to these inflammatory processes through additional upstream pro- and anti-inflammatory actions, but the exact pathways through which they act are not fully understood. Molecular structures of fatty acids and oxylipins were generated from lipidsmap.org.
(TIFF)

S2 Fig Diagnostic plots for all n = 28 genetic variants, showing the predicted association with T2D for the original top models (evaluating n = 47 oxylipin/fatty acid risk factors) with posterior probability ≥ 0.02, ordered by decreasing posterior probability. Plots on the left-hand side show the *q*-statistic for outliers, plots on the right-hand side show the Cook’s distance for influential points. Any genetic variant with a Cook’s distance larger than the median or a *q*-value larger than 10 is labelled with its rsid.
(TIFF)

S3 Fig Spearman correlation matrix of n = 81 putative fatty acids and oxylipin metabolites. Correlations were calculated accounting for survey weights. Values above the 99^th^ percentile for a given oxylipin or fatty acid were winsorized to the 99^th^ percentile value, and then all values were mean centered and scaled prior to computing correlations. Positive correlations are displayed in blue; negative correlations are displayed in red. Oxylipins and fatty acids are annotated with the putative general class of compound to which they belong, their measured mass-to-charge ratio (m/z), and retention time (RT) and ordered in the matrix based on these general groupings, with putative free fatty acids first (polyunsaturated fatty acids [PUFAs], monounsaturated fatty acids [MUFAs], saturated fatty acids [SFAs]), followed by oxylipin metabolites. If more than one identification is possible, we have listed all of them, separated by backslashes (e.g., HETE/EET).
(TIFF)
