# Supplementary material for: Evaluating associations of oxylipin metabolites and fatty acids with incident and prevalent type 2 diabetes in the Hispanic Community Health Study/Study of Latinos (HCHS/SOL)
Source: PLoS One. 2026 Oct 26;21(10):e0360656. doi: 10.1371/journal.pone.0360656 (PMC13704816; doi:10.1371/journal.pone.0360656)
Supplement: Supplementary file 1 [file pone.0360656.s006.pdf]

Plos One Review - PONE-D-26-25718

Evaluating associations of oxylipin metabolites and fatty acids with incident and prevalent type 2 diabetes in the Hispanic Community Health Study/Study of Latinos (HCHS/SOL)

Minor changes

Is the format in the Abstract where the sub-sections of Background, Methods, Results, Conclusions and Keywords are indicated standard for PLOS One? This presents as being slightly unconventional.

Please provide an initial clear explanation of the difference between incident and prevalent type 2 diabetes in the Introduction of the manuscript.

Line 203 - Was a particular version of SAS used? Any other detail that can be provided here?

Line 226 - Is this supposed to be anti-hyptertensive "medications"?

Line 235 - ….were missing less than 2 % of all observations? Is this missing words?

Line 250 - 254 - Consider breaking this into 2 sentences.

Line 437 - 439 - Is attrition here referring to patient participation attrition? Please clarify briefly.

Major changes

Line 144 - Please provide at least a succinct description of the oxylipin profiling approach rather than only referring to the Supplementary Methods. In particular, understanding how sampling was conducted to provide material for fatty acid/oxylipin profiling will be particularly helpful. Similarly, as per a subsequent comment, it would be helpful to understand if any analytical standard was used in the method set-up or validation to provide some basis for quantification.

Please add molecular structures of the important oxylipin/fatty acid structures provided - e.g. -adrenic, dihomo-gammalinolenic,

tricosanoic, lignoceric acids, DiHETrE (dihydroxyeicosatrienoic acid) - to enhance reader comprehension

It would also help reader comprehension to provide a schematic linking the most important metabolic/inflammatory pathways in type 2 disease progression with

- those important steps which give rise to the core fatty acids/oxylipins mentioned,
- and putative bioactive effects these fatty acids/oxylipine may elicit.

Line 344 - 350 - Are there any potential reasons for the differences in identification of oxylipins significantly associated with incident T2D between the FINRISK and HCHS/SOL studies based on the specifics of the analytical techniques (e.g. - LC-MS or other) used by each study? Some mention of this should be made.

Line 414 - 416 - Were any standard chemicals used to validate the analytical method that can provide some baseline for quantification?

The Conclusion section is overly short and lacking detail. This should be enhanced to provide a greater capstone to the manuscript. What are the next step, follow on studies that need to be completed?
