# Supplementary material for: Evaluating associations of oxylipin metabolites and fatty acids with incident and prevalent type 2 diabetes in the Hispanic Community Health Study/Study of Latinos (HCHS/SOL)
Source: PLoS One. 2026 Oct 26;21(10):e0360656. doi: 10.1371/journal.pone.0360656 (PMC13704816; doi:10.1371/journal.pone.0360656)
Supplement: Supplementary file 2 [file pone.0360656.s001.docx]

**Supplementary Methods**

*Bayesian model averaging multivariate Mendelian randomization (MR-BMA) statistical analysis*

To prioritize possible causal metabolites for type 2 diabetes (T2D), we used Bayesian model averaging Mendelian randomization (MR-BMA), an extension of multivariable MR that performs variable selection in a multivariable MR framework.[1] MR-BMA evaluates models with all possible combinations of provided “risk factors” as exposures (e.g., here, fatty acids and oxylipin metabolites), and computes the posterior probability that the model contains the true causal risk factors, for each model.[1] Subsequently, all posterior probabilities of models in which a specific risk factor was included are summed to generate a marginal inclusion probability for each risk factor, which is used to rank and prioritize the most likely causal risk factors.[1]

*Genetic instrument summary statistics for MR-BMA*

For the oxylipin and fatty acid “risk factors” (exposures), we used summary statistics from our previously published genome-wide association study (GWAS)[2] of the n=81 fatty acids and oxylipins in the HCHS/SOL cohort (n=11,584). For independent T2D outcome data, genetic associations with T2D were provided by the DIAMANTE Hispanic/Latino consortium, excluding HCHS/SOL participants (n T2D cases=23,541, n controls=37,434, all Hispanic/Latino participants).[3, 4]

*MR-BMA Risk factor and instrumental variable selection*

**S3a Table and S3b Table** show the lists of risk factors and instrumental variables considered and included in the final MR-BMA analysis. We restricted our list of candidate risk factors to all oxylipins and fatty acids with nominally significant (*p* < 5E-09) genetic associations, resulting in an initial list of 54 candidate risk factors. We used the mv_extract_exposures_local() function from the TwoSampleMR R package (v. 0.6.16)[5] to extract instrumental variables, with p_val_threshold = 5E-09, clumping LD threshold at r^2^= 0.001 and distance threshold=10000 KB, using the AMR 1000G reference population for calculating LD, identifying 28 independent variants as genetic instruments. The top variants for four oxylipins and fatty acids were not included in the final set of 28 identified independent variants (due to absence from the LD reference panel) and therefore these oxylipins and fatty acids were excluded from the candidate risk factor list since they lacked strong (*p* < 5E-09) instrumental variables, resulting in n=50 candidate risk factors. We used the mv_harmonize_data() function from the TwoSampleMR R package (v. 0.6.16)[5] to extract and harmonize the results from the T2D outcome summary statistics for these instrumental variables.

To evaluate multicollinearity between the 50 candidate oxylipins and fatty acids, we calculated Pearson correlation coefficients for each of the metabolites based on the 28 genetic variant effect estimates, standardized by the standard errors for the corresponding genetic variant-T2D associations. There were 3 pairs of metabolites with *r* > 0.985:

(1) Palmitoleic acid (m/z = 253.2182, RT=0.372) and Leukotriene/HpETE/DiHETE/Prostaglandin/Hepoxilin (m/z=335.222, RT=0.372)
(2) Putative_Known_Oxylipin (m/z = 349.2378, RT=0.349) and Putative_Novel_Oxylipin (m/z = 323.2224, RT = 0.343)
(3) Putative_Novel_Oxylipin (m/z = 365.2674, RT = 0.415) and Stearic acid (m/z = 283.2652 and RT = 0.415)

From these pairs, we randomly selected to exclude the following oxylipins and fatty acids from our candidate oxylipins and fatty acids: Leukotriene/HpETE/DiHETE/Prostaglandin/Hepoxilin (m/z=335.222, RT=0.372), Putative_Known_Oxylipin (m/z = 349.2378, RT=0.349), and Stearic acid (m/z = 283.2652 and RT = 0.415), resulting in n=47 candidate risk factors for MR-BMA analysis.

Following the example of Zuber et al,[1] after performing MR-BMA analysis for this list of 47 candidate risk factors and 28 corresponding instrumental variables, we identified all models with posterior probability > 0.02, which included the top 5 models. We then computed the Cook’s distance and q-statistic of all 28 variants to identify influential or outlying variants for each of these models. Variants with a q-statistic greater than 10 were considered potential outliers, and variants with Cook’s distance greater than the median of a central *F*-distribution with *d* and *n -d* degrees of freedom were considered potential influential points.[1] Variants rs4841132 and rs4988235 were each flagged as influential variants in one of these models (**S2 Figure**) and therefore we subsequently excluded these from our input list of genetic instruments, resulting in a final list of n=26 instrumental variables. Finally, we then excluded ten oxylipin and fatty acid risk factors from our candidate list, because after excluding rs4841132 and rs4988235 these risk factors no longer had any strong (*p <* 5E-09) genetic instruments. Our final lists of n=37 candidate risk factors and n=26 instrumental variables are provided in **S3a Table and S3b Table**, and the MR-BMA results we report are using these final inputs.

*MR-BMA analyses*

For MR-BMA analyses, we set the prior probability of selecting a risk factor to reflect three expected causal risk factors (but allowing up to twelve possible risk factors), the prior variance σ^2^=0.25, and 100,000 iterations in the shotgun stochastic search.[1, 6] We performed sensitivity analyses setting the prior probability to reflect one, two, four, and five expected causal risk factors, holding the prior variance constant at σ^2^=0.25. We computed Benjamini-Hochberg false discovery rate (FDR)-corrected p-values (q<0.05) for the top 10 most likely causal risk factors using an empirical permutation procedure, as previously described.[7, 8] This empirical permutation procedure recommends at least 100,000 permutations (generated by random sampling) for stability. Because of the long computational time anticipated for running 100,000 sequential repetitions, we ran parallel sets of permutations (25 repetitions per instance, 4000 parallel runs total), setting a different seed for each run. We concatenated the sets of permuted marginal inclusion probabilities (totaling 100,000 permutations) and verified that there were no duplicate permutation results across the total set of permutations. We then calculated the empirical and FDR-corrected *p* values from these results. All MR-BMA analyses were conducted in R (versions 4.4.0 and 4.5.0)[9] using the code and functions provided by the developers of this method (<https://github.com/verena-zuber/demo_AMD>).

**References**

1.	Zuber V, Colijn JM, Klaver C, Burgess S. Selecting likely causal risk factors from high-throughput experiments using multivariable Mendelian randomization. Nat Commun. 2020;11(1):29. Epub 2020/01/09. doi: 10.1038/s41467-019-13870-3. PubMed PMID: 31911605; PubMed Central PMCID: PMCPMC6946691.

2.	Downie CG, Highland HM, Alotaibi M, Welch BM, Howard AG, Cheng S, et al. Genome-wide association study reveals shared and distinct genetic architecture of fatty acids and oxylipins in the Hispanic Community Health Study/Study of Latinos. HGG Adv. 2025;6(1):100390. Epub 2024/12/07. doi: 10.1016/j.xhgg.2024.100390. PubMed PMID: 39644095; PubMed Central PMCID: PMCPMC11751521.

3.	Mahajan A, Spracklen CN, Zhang W, Ng MCY, Petty LE, Kitajima H, et al. Multi-ancestry genetic study of type 2 diabetes highlights the power of diverse populations for discovery and translation. Nat Genet. 2022;54(5):560-72. Epub 2022/05/14. doi: 10.1038/s41588-022-01058-3. PubMed PMID: 35551307; PubMed Central PMCID: PMCPMC9179018.

4.	Petty LE, Chen HH, Frankel EG, Zhu W, Downie CG, Graff M, et al. Large-scale multi-omics analyses in Hispanic/Latino populations identify genes for cardiometabolic traits. Nat Commun. 2025;16(1):3438. Epub 2025/04/11. doi: 10.1038/s41467-025-58574-z. PubMed PMID: 40210677; PubMed Central PMCID: PMCPMC11985957 Roche stock. L.S.E. is now an employee of Bristol Myers Squibb (BMS) and a holder of BMS stock. The remaining authors declare no competing interests.

5.	Hemani G, Zheng J, Elsworth B, Wade KH, Haberland V, Baird D, et al. The MR-Base platform supports systematic causal inference across the human phenome. Elife. 2018;7. Epub 2018/05/31. doi: 10.7554/eLife.34408. PubMed PMID: 29846171; PubMed Central PMCID: PMCPMC5976434.

6.	Servin B, Stephens M. Imputation-based analysis of association studies: candidate regions and quantitative traits. PLoS Genet. 2007;3(7):e114. Epub 2007/08/07. doi: 10.1371/journal.pgen.0030114. PubMed PMID: 17676998; PubMed Central PMCID: PMCPMC1934390.

7.	Zuber V, Gill D, Ala-Korpela M, Langenberg C, Butterworth A, Bottolo L, et al. High-throughput multivariable Mendelian randomization analysis prioritizes apolipoprotein B as key lipid risk factor for coronary artery disease. Int J Epidemiol. 2021;50(3):893-901. Epub 2020/11/02. doi: 10.1093/ije/dyaa216. PubMed PMID: 33130851; PubMed Central PMCID: PMCPMC8271202.

8.	Levin MG, Zuber V, Walker VM, Klarin D, Lynch J, Malik R, et al. Prioritizing the Role of Major Lipoproteins and Subfractions as Risk Factors for Peripheral Artery Disease. Circulation. 2021;144(5):353-64. Epub 2021/06/19. doi: 10.1161/CIRCULATIONAHA.121.053797. PubMed PMID: 34139859; PubMed Central PMCID: PMCPMC8323712.

9.	R Core Team. R: A Language and Environment for Statistical Computing. Vienna, Austria: R Foundation for Statistical Computing; 2025.
