# Supplementary material for: Evaluating associations of oxylipin metabolites and fatty acids with incident and prevalent type 2 diabetes in the Hispanic Community Health Study/Study of Latinos (HCHS/SOL)
Source: PLoS One. 2026 Oct 26;21(10):e0360656. doi: 10.1371/journal.pone.0360656 (PMC13704816; doi:10.1371/journal.pone.0360656)
Supplement: Supplementary file 3 [file pone.0360656.s008.docx]

We thank the reviewers for their thoughtful comments and suggestions, which have strengthened the quality of our manuscript. The revisions we have made in response to each comment are described below. Additionally, we realized that the fatty acid “tricosenic acid” had been mistakenly labeled as a “saturated fatty acid” rather than a “monosaturated fatty acid” in the annotations provided in the Supplementary Results. We have corrected this error and also provided a new Figure S3 (correlation plot) with tricosenic acid now grouped within the correct fatty acid grouping.

**Journal Requirements:**

When submitting your revision, we need you to address these additional requirements.

1. **Please ensure that your manuscript meets PLOS ONE's style requirements, including those for file naming. The PLOS ONE style templates can be found at https://journals.plos.org/plosone/s/file?id=wjVg/PLOSOne_formatting_sample_main_body.pdf and** [**https://journals.plos.org/plosone/s/file?id=ba62/PLOSOne_formatting_sample_title_authors_affiliations.pdf**](https://journals.plos.org/plosone/s/file?id=ba62/PLOSOne_formatting_sample_title_authors_affiliations.pdf)

We confirm that our manuscript meets PLOS ONE’s style requirements.

**2. We note that the grant information you provided in the ‘Funding Information’ and ‘Financial Disclosure’ sections do not match.**

**When you resubmit, please ensure that you provide the correct grant numbers for the awards you received for your study in the ‘Funding Information’ section.**

As described in more detail below, we have updated the Funding Information and Conflicts of Interest statements, which have been provided in our cover letter.

**3. Thank you for stating in your Funding Statement: [The Hispanic Community Health Study/Study of Latinos is a collaborative study supported by contracts from the**

**National Heart, Lung, and Blood Institute (NHLBI) to the University of North Carolina (HHSN268201300001I / N01- HC-65233), University of Miami (HHSN268201300004I / N01-HC-65234), Albert Einstein College of Medicine (HHSN268201300002I / N01-HC-65235), University of Illinois at Chicago (HHSN268201300003I / N01- HC-65236**

**Northwestern University), and San Diego State University (HHSN268201300005I / N01-HC-65237). The following Institutes/Centers/Offices have contributed to the HCHS/SOL through a transfer of funds to the NHLBI: National Institute on Minority Health and Health Disparities, National Institute on Deafness and Other Communication Disorders, National Institute of Dental and Craniofacial Research, National Institute of Diabetes and Digestive and Kidney Diseases, National Institute of Neurological Disorders and Stroke, NIH Institution-Office of Dietary Supplements. The DIAMANTE Consortium was supported through R01HL142302. Metabolite measurements were performed under approved ancillary study #2018.15, supported through R01HL147853. CGD was supported by T32HL129982. HMH was supported by R01HL147853, R01HL151152, R56HG010297. CLA was supported by R01HL147853.]. Please provide an amended statement that declares *all* the funding or sources of support (whether external or**

**internal to your organization) received during this study, as detailed online in our guide for authors at http://journals.plos.org/plosone/s/submit-now. Please also include the statement “There was no additional external funding received for this study.” in your updated Funding Statement. Please include your amended Funding Statement within your cover letter. We will change the online submission form on your behalf.**

We have confirmed that all funding sources are provided in the Funding Statement, and have added the line “There was no additional external funding received for this study” to the statement.

**4. Please remove any funding-related text from the manuscript. Please note that funding information should not appear in any section or other areas of your manuscript. We will publish the funding statement that is present in the Financial Disclosure section of the online submission form.**

We have removed all funding related text from the manuscript. Our revised Funding Statement is provided in the cover letter.

**5. Thank you for stating the following in the Competing Interests section:**

**[Mohit Jain and Tao Long are employees of Sapient Bioanalytics. All other authors have nothing to disclose.].**

**We note that one or more of the authors are employed by a commercial company: Sapient Bioanalytics.**

1. **Please provide an amended Funding Statement declaring this commercial affiliation, as well as a statement regarding the Role of Funders in your study. If the funding organization did not play a role in the study design, data collection and analysis, decision to publish, or preparation of the manuscript and only provided financial support in the form of authors' salaries and/or research materials, please review your statements relating to the author contributions, and ensure you have specifically and accurately indicated the role(s) that these authors had in your study. You can update author roles in the Author Contributions section of the online submission form. Please also include the following statement within your amended Funding Statement. “The funder provided support in the form of salaries for authors [insert relevant initials], but did not have any additional role in the study design, data collection and analysis, decision to publish, or preparation of the manuscript. The specific roles of these authors are articulated in the ‘author contributions’ section.” If your commercial affiliation did play a role in your study, please state and explain this role within your updated Funding Statement.**

We have amended the Funding Statement to include the following information about the contributions of Sapient Bioanalytics to the study:

“Metabolite measurements were performed under approved HCHS/SOL ancillary study #2018.15, which was supported through R01HL147853. Sapient Bioanalytics was contracted to generate these oxylipin and fatty acid metabolite data. Sapient Bioanalytics also provided support in the form of salaries for MJ and TL, who are employees of the company. The specific roles of these authors are articulated in the ‘Author Contributions’ section. Sapient Bioanalytics did not play any additional role in decision to publish.”

2. **Please also provide an updated Competing Interests Statement declaring this commercial affiliation along with any other relevant declarations relating to employment, consultancy, patents, products in development, or marketed products, etc. Within your Competing Interests Statement, please confirm that this commercial affiliation does not alter your adherence to all PLOS ONE policies on sharing data and materials by including the following statement: "This does not alter our adherence to PLOS ONE policies on sharing data and materials.” (as detailed online in our guide for authors http://journals.plos.org/plosone/s/competing-interests). If this adherence statement is not accurate and there are restrictions on sharing of data and/or materials, please state these. Please note that we cannot proceed with consideration of your article until this information has been declared. Please include both an updated Funding Statement and Competing Interests Statement in your cover letter. We will change the online submission form on your behalf.**

We have amended the Competing Interests Statement to include this statement, as follows:

“Mohit Jain and Tao Long are employees of Sapient Bioanalytics, which performed the oxylipin and fatty acid metabolite profiling analyses to generate the metabolite data included in this analysis. This does not alter our adherence to PLOS One policies on sharing data and materials. All other authors have nothing to disclose.”

**6. For studies involving third-party data, we encourage authors to share any data specific to their analyses that they can legally distribute. PLOS recognizes, however, that authors may be using third-party data they do not have the rights to share. When third-party data cannot be publicly shared, authors must provide all information necessary for interested researchers to apply to gain access to the data. (https://journals.plos.org/plosone/s/data-availability#loc-**

**acceptable-data-access-restrictions) For any third-party data that the authors cannot legally distribute, they should include the following information in their Data Availability Statement upon submission:**

**1) A description of the data set and the third-party source|**

**2) If applicable, verification of permission to use the data set**

**3) Confirmation of whether the authors received any special privileges in accessing the data that other researchers would not have**

**4) All necessary contact information others would need to apply to gain access to the data.**

We have revised our Availability of Data statement as follows:

“The HCHS/SOL datasets, which include a baseline cohort of 16,415 self-identified Hispanic/Latino individuals recruited across four centers in the United States, are available through approved requests with the HCHS/SOL study. The policies for such data requests are described at <https://sites.cscc.unc.edu/hchs/>. The authors did not have any special privileges in accessing the data that other future researchers would not have.”

**7. Your ethics statement should only appear in the Methods section of your manuscript. If your ethics statement is written in any section besides the Methods, please delete it from any other section.**

We have removed the additional ethics statement and confirm that all ethics statement information is now provided only in the Methods section, lines 141-145:

“The HCHS/SOL was approved by institutional review boards at each study center as well as the HCHS/SOL Data Coordinating Center at the University of North Carolina at Chapel Hill and was conducted in accordance with the Declaration of Helsinki.(31) All participants gave written informed consent prior to the collection of any data.(31)”

**8. We note you have included a table to which you do not refer in the text of your manuscript. Please ensure that you refer to Tables 2 and 3 in your text; if accepted, production will need this reference to link the reader to the Tables.**

Tables 2 and 3 are clearly referred to in lines 427 and 428 of the manuscript text:

“…In MR-BMA analysis of n=37 heritable oxylipins and fatty acids for which at least one genetic instrument could be identified, a diHETrE oxylipin (m/z = 337.2361, RT=0.389) was prioritized as the top-ranked causal risk factor for T2D (margin inclusion probability = 0.601, *p*=2.80E-04, FDR-corrected *p*=1.04E-02, model averaged causal effect = -0.342) (**Tables 2 and 3**).”

**9. Please include captions for all your Supporting Information files at the end of your manuscript, and update any in-text citations to match accordingly. Please see our Supporting Information guidelines for more information:**

**http://journals.plos.org/plosone/s/supporting-information.**

We have provided captions for the three Supporting Figures at the end of the manuscript.

**If the reviewer comments include a recommendation to cite specific previously published works, please review and evaluate these publications to determine whether they are relevant and should be cited. There is no requirement to cite these works unless the editor has indicated otherwise. Please review your reference list to ensure that it is complete and correct. If you have cited papers that have been retracted, please include the rationale for doing so in the manuscript text, or remove these references and replace them with relevant current references. Any changes to the reference list should be mentioned in the rebuttal letter that accompanies your revised manuscript. If you need to cite a retracted article, indicate the article’s retracted status in the References list and also include a citation and full reference for the retraction notice.**

Not applicable; the reviewers have not recommended specific references.

**Additional Editor Comments:**

**As also indicated by the two reviewers please made these minor changes**

**1. Analytical Methodology & Reporting Standards: Clarify the oxylipin and fatty acid profiling methodology in the main text (including sampling, LC-MS setup/chromatography details, and standard chemical validation). Address the discrepancy regarding reported retention times (<1 minute vs. standard HPLC-MS), detail sample storage/extraction**

**protocols (e.g., use of antioxidants or internal standards), and explain analytical differences when comparing findings to existing literature (e.g., FINRISK).**

We have addressed these requests, which we describe in more detail below in our response to the comments from the reviewers.

**2. Confounding Variables & Subgroup Analyses: Address potential confounding factors by considering stratification for statin and aspirin use and adjusting or discussing the impact of plasma triglyceride levels on oxylipin content (e.g., DiHETrE). Additionally, clarify specific manuscript details such as patient attrition, the definition/distinction of incident vs. prevalent T2D in the Introduction, and ensure Table 1 reports smoking status statistics as noted in the text.**

We have addressed these requests, which we describe in more detail below in our response to the comments from the reviewers.

**3. Presentation, Visual Aids, and Textual Enhancements: Enhance reader comprehension by adding molecular structures for key fatty acids/oxylipins and providing a schematic/diagram linking relevant metabolic/inflammatory pathways to the study's core markers. Furthermore, expand the Conclusion section to provide a comprehensive discussion of next steps and future follow-on studies.**

We have addressed these requests, which we describe in more detail below in our response to the comments from the reviewers.

**Reviewers' comments:**

**Reviewer #1: Minor changes**

1. **Is the format in the Abstract where the sub-sections of Background, Methods, Results, Conclusions and Keywords are indicated standard for PLOS One? This presents as being slightly unconventional.**

PLOS One does not have specific requirements specifying whether the Abstract should be structured or non-structured (<https://journals.plos.org/plosone/s/submission-guidelines>), and we have seen examples of both abstract styles in recent PLOS One publications. We have removed the keywords section, since this does not appear to be a PLOS One requirement. However, we will defer to the editor on additional formatting changes and convert to a non-structured abstract style if requested.

2. **Please provide an initial clear explanation of the difference between incident and prevalent type 2 diabetes in the Introduction of the manuscript.**

We have added the following sentence to the Introduction of the manuscript to differentiate between incident and prevalent type 2 diabetes:

Lines 119-124: “Leveraging the Hispanic Community Health Study/Study of Latinos (HCHS/SOL), a population with a high burden of diabetes,(27-29) we examined the relationships between oxylipins and fatty acids and T2D, including cross-sectional associations between oxylipins and fatty acids measured at study visit 1 and established T2D at study visit 1 (prevalent T2D), and prospective associations between oxylipins and fatty acid metabolites measured at study visit 1 and T2D diagnosed by study visit 2 among individuals free of T2D at baseline (incident T2D).”

3. **Line 203 - Was a particular version of SAS used? Any other detail that can be provided here?**

We have updated line 265-266 to specify that SAS version 9.4 was used for PC analyses: “PC analyses were conducted in SAS (version 9.4, SAS Institute).”

4. **Line 226 - Is this supposed to be anti-hyptertensive "medications"?**

To avoid repetition, we had previously written “medications” only once at the end of this clause (lines 226-227): “were much more likely to use anti-hypertensive (survey-weighted percentage (SE) 54.3% (1.4)), statin (32.4% (1.4)), and aspirin (40.6% (1.5)) **medications** than participants without prevalent T2D (**Table 1**).”

However, since we see this has caused confusion, we have revised the text in the manuscript as follows:

Lines 291-293: “…were much more likely to use anti-hypertensive **medications** (survey-weighted percentage (SE) 54.3% (1.4)), statins (32.4% (1.4)), and aspirin (40.6% (1.5)) than participants without prevalent T2D (**Table 1**)”

5. **Line 235 - ….were missing less than 2 % of all observations? Is this missing words?**

This sentence was not missing any words. This section describes the metabolite-level

missingness, i.e. for each of the 81 metabolites, how many individuals had missing values for the given metabolite. 92.6% of the 81 metabolites had fewer than 2% of all values missing.

However, to try to improve the clarity of this sentence, we have revised the manuscript text as follows:

Lines 299-301: “The percent missingness for each of the 81 oxylipins and fatty acids measured at visit 1 ranged from 0% to 23.1%, though most metabolites (92.6%) were missing less than 2% of their values.”

6. **Line 250 - 254 - Consider breaking this into 2 sentences.**

We have revised the manuscript text as follows:

Lines 315-319: “However, we note that another fatty acid with similar m/z and RT also annotated as lignoceric acid (m/z=367.3577, RT=0.496) had a significant positive association with prevalent T2D (OR [95% CI] = 1.09 [1.01, 1.17], FDR-corrected *p*=3.20E-02). This positively-associated lignoceric acid metabolite was only modestly correlated (ρ=0.26) with the other negatively-associated lignoceric acid metabolite (m/z=367.3576, RT=0.469).”

7. **Line 437 - 439 - Is attrition here referring to patient participation attrition? Please clarify briefly.**

We have tried to clarify that this is referring to patient participation attrition by revising the manuscript text as follows:

Lines 552 – 555: “Finally, participant attrition at subsequent study visits over the course of longitudinal follow-up resulted in progressively smaller sample sizes at study visits 2 and 3, limiting power to detect any associations with small effects.”

**Major changes**

8. **Line 144 - Please provide at least a succinct description of the oxylipin profiling approach rather than only referring to the Supplementary Methods. In particular, understanding how sampling was conducted to provide material for fatty acid/oxylipin profiling will be particularly helpful. Similarly, as per a subsequent comment, it would be helpful to understand if any analytical standard was used in the method set-up or validation to provide some basis for quantification.**

We thank the reviewer for this suggestion. We have now moved the description of the metabolite profiling from the Supplementary Methods to the main text (see the revised “Oxylipin profiling and annotation” subsection in the Methods, lines 147-202). With regards to analytical standards: a panel of isotopically labeled internal standards (including ¹³C₅¹⁵N₁-glutamate, 16:0-d31-18:1 PC, 14:0-16:1-14:0 d5 TG, arachidonic acid-d11, and CUDA) was used throughout data collection for quality control to monitor extraction efficiency, matrix effects, mass accuracy, and instrument sensitivity/drift across batches. These standards support relative quantification and batch monitoring; they were not used to derive absolute concentrations, and all oxylipin and fatty acid values reported in this study are relative abundance measures. We have made this explicit in the Methods.

9. **Please add molecular structures of the important oxylipin/fatty acid structures provided - e.g. -adrenic, dihomo- gammalinolenic, tricosanoic, lignoceric acids, DiHETrE (dihydroxyeicosatrienoic acid) - to enhance reader comprehension. It would also help reader comprehension to provide a schematic linking the most important metabolic/inflammatory pathways in type 2 disease progression with those important steps which give rise to the core fatty acids/oxylipins mentioned, and putative bioactive effects these fatty acids/oxylipine may elicit.**

We thank the reviewer for this suggestion. We have provided a supplementary figure outlining the primary biosynthetic pathways for fatty acids and oxylipin derivatives and reported inflammatory relationships to glycemic dysregulation. This figure includes molecular structures for the major fatty acids and an example of a DiHETrE oxylipin, and indicates whether certain classes of oxylipins are known to have primarily pro-inflammatory or anti-inflammatory effects, as reported in previous studies. However, we note that the precise inflammatory actions of many oxylipins may vary based on the receptors they interact with (PMID: 28508285), and the inflammatory actions of the numerous putatively novel oxylipins included in our study are unknown. Therefore, the inflammatory pathways and bioactive effects that these metabolites may elicit are unknown, and we feel that speculating on their roles is beyond the scope of our results.

We refer to this figure on lines 103-105: “A schematic summarizing several reported primary biosynthetic pathways of fatty acids and oxylipins(14-16) and their reported bioactive inflammatory roles in glycemic dysregulation(1, 2, 17, 18) is shown in **S1 Figure.**”

10. **Line 344 - 350 - Are there any potential reasons for the differences in identification of oxylipins significantly associated with incident T2D between the FINRISK and HCHS/SOL studies based on the specifics of the analytical techniques (e.g. - LC-MS or other) used by each study? Some mention of this should be made.**

Both studies’ plasma samples were profiled by Sapient Bioanalytics. However, in the Tuomisto et al study (PMID: 35361620), FINRISK samples were profiled on a eicosanoid-focused directed non-targeted LC-MS platform/chemical-networking method. Differences between these platforms in chromatographic separation, ionization efficiency, and the annotation/chemical-networking pipeline used to assign compound identities are a plausible contributor to differences in which oxylipins are identified as associated with incident T2D across cohorts, which we have added to the Discussion section:

Lines 457-463: “However, in this FINRISK study, plasma samples were profiled using a different, eicosanoid-focused directed non-targeted LC-MS/chemical networking method (Sapient Bioanalytics) than the method used for our current study. Differences in chromatographic separation, ionization efficiency, and annotation/chemical-networking pipeline used to assign compound identities are thus plausible contributors to differences in the incident T2D-metabolite associations identified across these cohorts.”

11. **Line 414 - 416 - Were any standard chemicals used to validate the analytical method that can provide some baseline for quantification?**

We have now provided a longer description of the metabolite profiling method in the main Methods instead of the Supplementary Methods, which includes discussion of standard chemicals used in the analytical process. As described above, a panel of isotopically labeled internal standards was used to validate and monitor the analytical method throughout data collection. These standards support quality control and relative quantification; they do not provide a basis for absolute quantification, and we have clarified in the Methods that all reported oxylipin and fatty acid values are relative abundance measures.

12. **The Conclusion section is overly short and lacking detail. This should be enhanced to provide a greater capstone to the manuscript. What are the next step, follow on studies that need to be completed?**

We have added the following sentences to the Conclusion section, lines 561-566:

“Future studies should conduct similar analyses in other diverse populations to improve understanding of the generalizability of our results across populations. Furthermore, future efforts to annotate and characterize these oxylipin metabolites, particularly putatively novel oxylipins, will be important to better understand the biological pathways and disease processes to which these metabolites may contribute.”

**Reviewer #2:**

**Point 1) The Supplementary Methods state that the analytical method used is the same as the one previously used and described in Articles 1 and 2 of the Supplementary Text. However, the retention time data reported in the Supplementary Tables are all below one minute, suggesting a direct injection approach, while in the cited works, the retention times reported for the analytes are consistent with fast chromatography, which however requires the use of a column. Clarifying this aspect is also essential for the interpretation of potentially confounding data, given that most oxylipins have a similar m/z, even though they could be distinguished chromatographically. This is because the analytical approach used makes it difficult to obtain characteristic and identifying fragmentations, as is the case with HPLC-MS analyses involving MRM with a triple quadrupole. Furthermore, details on the plasma storage methods from the time of collection and the extraction procedures (addition or not of antioxidants such as BHT, addition of deuterated internal standards, SPE, etc.) are missing.**

We thank the reviewer for flagging this inconsistency, which reflects a citation error on our part rather than an error in the underlying data. The sub-one-minute retention times are correct and expected: HCHS/SOL profiling was performed on a rapid resolution LC-MS (rLC-MS) platform, distinct from the conventional eicosanoid/bioactive-lipid LC-MS assay we had previously cited (which uses longer reverse-phase chromatographic gradients on different instrumentation). The rLC-MS platform still separates analytes chromatographically but does so via rapid nano-valve switching and a multi-tiered isocratic elution on a custom mixed-mode column, achieving a per-sample cycle time on the order of one minute. We have corrected the Methods and citations to describe this platform accurately (see Watrous et al. 2025, bioRxiv; DOI 10.1101/2025.08.10.669154, added as reference 33). On plasma storage and extraction: plasma was isolated and frozen, transported on dry ice, then thawed, aliquoted, and stored at –80°C until processing. Extraction used protein precipitation with an acetonitrile:methanol (65:35) solvent containing 0.5% formic acid, with isotopically labeled internal standards added at this step; antioxidants such as BHT were not added, and extraction did not use solid-phase extraction (SPE). We have added these details to the Methods.

**Unfortunately, the study has a number of limitations that make it applicable only to the cohort already examined, which, although very large, consists exclusively of the Hispanic population of the United States. Absolute data on analyte concentrations are completely missing, making it impossible to replicate the study in other cohorts. This is also because this study stems from a previous study that examined the associations between genetic profile and fatty acids and oxylipins in the HCHS/SOL cohort, which had identified both shared and specific genetic bases for fatty acid and oxylipin levels in the Hispanic population. The limitations of the analytical method in fully characterizing oxylipins make comparison with other data reported in the literature using other analytical platforms and referring to predominantly European cohorts difficult. These limitations are noted and justified in the study. However, some aspects regarding the potential impact of confounding factors need further clarification.**

**- Both statins and aspirin have a strong impact on lipid metabolism and consequently on the plasma content of oxylipins and fatty acids. Much depends on the dosage and duration of treatment with these drugs. Therefore, a possible difference in lipid metabolism between healthy subjects and those with T2D who were not treated with statins or aspirin should be considered. The sample size could perhaps allow for this stratification.**

Recognizing the possible impact of medications on lipid metabolism, fatty acids, and oxylipins, we did adjust for reported statin, aspirin, and anti-hypertensive medication use in our primary analyses. However, as the reviewer has noted, since dosage and duration information was not also available, it is possible that residual confounding may remain.

As additional analyses, we repeated our analyses restricting to individuals who did not report taking any of these three medications. As additional sensitivity analyses, we also performed these analyses in single non-medication-restricted samples (i.e., individuals not taking statins, still adjusting for any aspirin and anti-hypertensive use; individuals not taking aspirin, still adjusting for statin and anti-hypertensive use; individuals not taking anti-hypertensives, still adjusting for aspirin and statin use). In these restricted samples, effects were consistent in direction and generally consistent in magnitude, though there were small numerical effect estimate changes. However, we believe these are primarily related to the reduced sample size. We report these results in the Results section:

Metabolite associations with prevalent T2D, lines 331-338: “When we restricted to individuals who did not report taking any of the three aspirin, statin, and anti-hypertensive medications (n=9718, n prevalent T2D=958), effect estimates remained directionally consistent with small numerical changes (median effect estimate difference [IQR]=-2.27E-02 [-5.28E-02, 1.44E-02]), which is likely a result of the smaller sample size, since effect estimate differences were smaller in single medication-restricted analyses with sample sizes closer to the primary analysis sample (e.g. among non-statin users, the largest non-medication-restricted sample with n=13460, median effect estimate difference [IQR] = -5.34E-03 [-1.72E-02, 1.34E-02]).”

Associations between prevalent T2D at visit 1 and visit 2-measured fatty acid levels, lines 362-367: “When we restricted to individuals who did not report taking any aspirin, statin, and anti-hypertensive medications at visit 1 and also did not report taking any statin and anti-hypertensive medications at visit 2 (n=4601, n prevalent T2D=349), effect estimates remained directionally consistent, though with some modest numerical changes, again likely reflecting the smaller sample size (median effect estimate difference [IQR]=-3.73E-02 [-7.26E-02, -3.07E-03]).”

Metabolite associations with incident T2D, lines 388-396: “As in the prevalent T2D analyses, when we restricted to individuals who did not report taking any of the aspirin, statin, and anti-hypertensive medications (n=5958, n T2D events = 799), effect estimates remained generally directionally consistent with small numerical changes (median effect estimate difference [IQR]=-1.56E-02 [-3.87E-02, 9.54E-01]), again likely reflecting the lower sample size, as effect estimate differences were lower in single non-medication-restricted analyses with sample sizes closer to the primary analysis sample size (e.g. among non-statin users, n=7,990, the largest single non-medication-restricted sample, median effect estimate difference [IQR]=-4.26E-04 [-7.28E-03, 9.55E-01]).”

**- Oxylipin content, and therefore also DiHeTrE, is directly correlated with triglyceride content: the study makes no reference to the possible impact of plasma triglyceride content.**

We thank the reviewer for this suggestion. We have run sensitivity analyses additionally adjusting for triglycerides and calculated the difference in effect estimates between our original model and these new models. Adjusting for triglycerides did not meaningfully impact the direction or magnitude of effect of our effect estimates. We have also reported on triglyceride values in Table 1. We report these results in the Results section:

Metabolite associations with prevalent T2D, lines 328-331: “Likewise, effect estimates were also generally consistent in models also adjusted for triglycerides (median beta effect estimate difference [IQR] comparing the original model to a model additionally adjusting for continuous triglycerides= -3.41E-03 [-9.37E-03, 4.21E-03], sample size n=15,126).”

Associations between prevalent T2D at visit 1 and visit 2-measured fatty acid levels, lines 359-362: “Similarly, additionally adjusting for triglycerides at visit 2 also had minimal impacts on results (median effect estimate difference [IQR] comparing the original model and a model additionally adjusted for continuous triglycerides =1.59E-04 [-4.05E-03, 7.25E-03], n=9,113, n prevalent T2D=2,150).”

Metabolite associations with incident T2D, lines 385-388: “Effect estimates were also generally consistent in models adjusted for triglycerides (median beta effect estimate difference [IQR] comparing original model to model additionally adjusting for continuous triglycerides value =-2.48E-03 [-5.63E-03, 1.03E-03]; n=8540, n T2D events=1375).”

**- The text states that "Results were consistent in models also adjusted for smoking status." However, Table 1 does not report the percentage of smokers.**

We thank the reviewer for pointing this out, we apologize for this oversight. We have now added the percentage of current smokers to Table 1. For consistency, we have now also reported the median effect estimate difference comparing the original model to the smoking-adjusted model in the Results section:

Metabolite associations with prevalent T2D, lines 325-328: “In sensitivity analyses, effect estimates were consistent in models also adjusted for smoking status (median beta effect estimate difference across all 81 metabolites comparing the original model to a model also including current smoking status [interquartile range]= -2.93E-04 [-7.09E-04, 7.96E-05], sample size n=15,120).”

Associations between prevalent T2D at visit 1 and visit 2-measured fatty acid levels, lines 356-359: “As in the prevalent T2D cross-sectional associations, additionally adjusting for current smoking status at visit 2 had minimal impacts on the effect estimates (median beta effect estimate difference [IQR] comparing the original model to a model adjusting for smoking =2.25E-03 [1.24E-04, 3.78E-03], n=9,111, n=1,331 current smokers).”

Metabolite associations with incident T2D, lines 382-385: “In sensitivity analyses, effect estimates were consistent in models also adjusted for smoking (median beta effect estimate difference [IQR] comparing the original model to a model also including current smoking status = 1.34E-03 [1.19E-04, 2.82E-03], sample size n=8,539, n T2D events=1,375).”
